# On the Prognosis and Treatment of Diabetes

**Published:** 1855-01

**Authors:** 


On the Prognosis and Treatment of Diabetes. By Professor Schutzenberger.—The conclusions, &c., drawn by the author are as follows:—

1. Glucosuria is not an incurable disease; if the disposition to relapse cannot be disputed, it is, however, possible, with perseverance, not only to banish the sugar from the urine, but gradually to bring the patient to bear a mixed regimen into which farinaceous matters enter in considerable proportion without causing relapses.

2. The quantity of glucose passed by the patients is sensibly proportional to the quantity of farinaceous matters taken as food, and it is possible to infer departures from the prescribed diet from the increased amount of glucose in the urine. The quantity of urine is perceptibly increased in proportion as a larger bulk of fluid is ingested, and that of the glucose is similarly influenced by an augmented consumption of farinaceous principles.

3. Regimen should incontestibly have a prominent position in the treatment of glucosuria. Milk, fatty matters, butter, oil, eggs, and meat, ought to form the bulk of the patient's food.

4. Total abstinence from farinaceous substances seems necessary to cause a complete disappearance of glucose from the urine.

5. Small quantities of bread (between three and four ounces a day) are generally well borne, and do not cause the reappearance of the sugar in the urine when the glucose has once ceased to be produced.

6. The powers of assimilation gradually increase, and it is possible, by means of chemical analysis, to demonstrate the point at which it is necessary to stop.

7. Regimen is powerfully seconded by certain medicines, and more especially by the use of opium in progressive doses, and of alkaline drinks:—in this disease the tolerance of opium is very great.

8. The glucose is undoubtedly produced in the digestive organs, but it is generally again removed by absorption, so that the solid fæces do not contain a trace of it.

9. Purgatives may diminish the quantity of glucose in the urine by removing with the fæcal matters a greater or less amount which would have been eliminated with that secretion.—*Bulletino delle Scienze Mediche de Bologna. January*, 1854.

